# Chemical Differentiation of* Dendrobium officinale* and* Dendrobium devonianum* by Using HPLC Fingerprints, HPLC-ESI-MS, and HPTLC Analyses

**DOI:** 10.1155/2017/8647212

**Published:** 2017-07-10

**Authors:** Zi Ye, Jia-Rong Dai, Cheng-Gang Zhang, Ye Lu, Lei-Lei Wu, Amy G. W. Gong, Hong Xu, Karl W. K. Tsim, Zheng-Tao Wang

**Affiliations:** ^1^The Ministry of Education Key Laboratory for Standardization of Chinese Medicines, The State Administration of Traditional Chinese Medicine Key Laboratory for New Resources and Quality Evaluation of Chinese Medicines, Institute of Chinese Materia Medica, Shanghai University of Traditional Chinese Medicine, Shanghai 201203, China; ^2^Shanghai R&D Center for Standardization of Chinese Medicines, Shanghai 201203, China; ^3^Longling County Research Institute of Dendrobium, Yunnan 678300, China; ^4^Division of Life Science and Center for Chinese Medicine, The Hong Kong University of Science and Technology, Clear Water Bay Road, Kowloon, Hong Kong

## Abstract

The stems of* Dendrobium officinale* Kimura et Migo (Dendrobii Officinalis Caulis) have a high medicinal value as a traditional Chinese medicine (TCM). Because of the limited supply,* D. officinale* is a high priced TCM, and therefore adulterants are commonly found in the herbal market. The dried stems of a closely related* Dendrobium* species,* Dendrobium devonianum* Paxt., are commonly used as the substitute; however, there is no effective method to distinguish the two* Dendrobium* species. Here, a high performance liquid chromatography (HPLC) method was successfully developed and applied to differentiate* D. officinale* and* D. devonianum* by comparing the chromatograms according to the characteristic peaks. A HPLC coupled with electrospray ionization multistage mass spectrometry (HPLC-ESI-MS) method was further applied for structural elucidation of 15 flavonoids, 5 phenolic acids, and 1 lignan in* D. officinale*. Among these flavonoids, 4 flavonoid C-glycosides were firstly reported in* D. officinale*, and violanthin and isoviolanthin were identified to be specific for* D. officinale* compared with* D. devonianum*. Then, two representative components were used as chemical markers. A rapid and reliable high performance thin layer chromatography (HPTLC) method was applied in distinguishing* D. officinale* from* D. devonianum*. The results of this work have demonstrated that these developed analytical methods can be used to discriminate* D. officinale* and* D. devonianum* effectively and conveniently.

## 1. Introduction

*Dendrobium* is one of the largest genera among Orchidaceae family plants with more than 1400 species distributed all over the world, and 76 species are found in China [1]. Some* Dendrobium* species have long been used as precious tonic herbs (Chinese name: Shi-Hu) in traditional Chinese medicine (TCM), which aims to promote the production of body fluids, benefit the stomach, moisten the lungs, and relieve cough [2].* Dendrobium officinale* Kimura et Migo (Dendrobii Officinalis Caulis) is one of the most expensive species among medicinal* Dendrobium* species. TCM practitioners have widely used* D. officinale* stems to treat hyperglycemia, hyperlipidemia, chronic gastritis, and dim eyesight and strengthen immunity, as well as an anticancer and antiaging agent, clinically [3].* D. officinale* stem is often dried directly or processed into a spiral shape while drying, which is named Tie-Pi-Feng-Dou in Chinese, and is officially recorded in the Chinese Pharmacopoeia [4].

Due to similar morphological characteristics and producing sites, Tie-Pi-Feng-Dou is often confused or mix-used with Zi-Pi-Feng-Dou processed by another* Dendrobium* species,* D. devonianum*, which is mainly produced in Yunnan province.* D. devonianum* stem is recorded officially in local production sites with the same clinical efficiency and usage as Tie-Pi-Feng-Dou [5]. Zi-Pi-Feng-Dou is even purposely named Tie-Pi-Feng-Dou and sold on the market by illegal business behaviors for more profits. This adulteration problem could result in highly variable and inconsistent therapeutic effects in clinical application, which therefore creates a potential health hazard [6]. The stems of* D. officinale* and* D. devonianum* have very similar morphological resemblance, and indeed it is difficult to differentiate them just by their appearance. Pharmacological experts and clinical practitioners are often bewildered by these two species when putting their crude drug in use. Unfortunately, there is so far no effective method to identify and distinguish Tie-Pi-Feng-Dou and Zi-Pi-Feng-Dou, that is,* D. officinale* from* D. devonianum*.

Chemical analyses have shown that polysaccharides, phenols, flavonoids, and coumarins are the main compounds isolated from the stems of* D. officinale* [7] and* D. devonianum* [5, 8]. Polysaccharide is the richest ingredient and is considered to be responsible for the pharmacological activities; however, the content of polysaccharide has no obvious difference in* D. officinale* and* D. devonianum* stems [9]. Flavonoids are another important compound in* D. officinale* and* D. devonianum* due to their potential antioxidant, anti-inflammatory, and anticancer properties.* D. officinale* is rich in the flavonoid C-glycoside [7], while* D. devonianum* is rich in the flavonoid O-glycoside [10, 11]. HPLC studies focusing on naringenin content [12], fingerprint patterning [13–15], and fragmentation pathways of flavonoids [16] in* D. officinale* did not provide any distinction between* D. officinale* and* D. devonianum.*

Chromatographic fingerprint comprises one of the important strategies for the quality control of herbal medicines, including identification, and discrimination of some closely related species since they can provide abundant information of complex chemical constituents [17, 18]. Here, we aimed to develop an effective HPLC method for fingerprinting analysis, which was being applied to distinguish the stems of* D. officinale* and* D. devonianum*. Specific chemicals, identified by HPLC-ESI-MS^n^, in* D. officinale* were used as indicative markers for proper identification. In addition, the TLC method of using two flavonoids as chemical markers was developed in differentiating* D. officinale* from* D. devonianum* stems.

## 2. Materials and Methods

### 2.1. Plant Materials, Chemicals, and Reagents

Twenty batches of stems of* D. officinale* and* D. devonianum* were collected from different parts of China from 2014 to 2016. Five batches of* D. officinale* (S1–S5) were collected from Mang City (Yunnan province), three batches of* D. officinale* (S6–S8) were collected from Liancheng County (Fujian province), and two batches of* D. officinale* (S9-S10) were collected from Yueqing County (Zhejiang province). Ten batches of* D. devonianum* (S11–S20) were collected from Longling County (Yunnan province). All the plant samples were authenticated by one of the authors, Professor Hong Xu. Voucher specimens were deposited at the Institute of Chinese Materia Medica, Shanghai University of TCM, Shanghai, China. Fresh stems were collected and processed into the marketable form, called Feng-Dou. Herbs were ground into powder before the analysis.

Violanthin, isoviolanthin, and vitexin-2′′-O-*β*-D-glucopyranoside were isolated from the stems of* D. officinale*. Schaftoside and isoschaftoside were purchased from Chengdu Mansite Pharmaceutical Co., Ltd. (Chengdu, China). Rutin and naringenin were obtained from Shanghai R&D Center for Standardization of Chinese Medicine (Shanghai, China). The purities of the abovementioned chemicals were determined to be more than 98% by normalization of the peak areas detected by HPLC-DAD, as well as by HPLC/MS. HPLC-grade acetonitrile and methanol were purchased from Fisher Chemicals Co. (New Jersey, USA). Analytical-grade ethyl acetate, butanone, and formic acid were purchased from Sinopharm Chemical Reagent Co., Ltd. (Shanghai, China). Deionized water was prepared by a Millipore Milli-Q Plus system (Millipore, Bedford, USA) and used in all experiments.

### 2.2. Preparation of Standard and Sample Solutions

The references, including violanthin, isoviolanthin, vitexin-2′′-O-*β*-D*-*glucopyranoside, schaftoside, isoschaftoside, rutin, and naringenin, were accurately weighed and dissolved in methanol to obtain a stock standard solution at a concentration of 1 mg/mL, respectively. The solutions were stored at 4°C and kept in a dark place. The processed dried stems of* D. officinale* and* D. devonianum* were ground into powder and then passed through a 50-mesh sieve. Powders (2 g) were accurately weighed and immersed in 100 mL of methanol and extracted in an ultrasonic bath at 25°C for 30 min. The supernatants were divided into two equal portions and concentrated, respectively,* in vacuo* to obtain two brownish-black colored residues. The first residue was redissolved in 1 mL of methanol and filtered through a 0.45 *μ*m membrane as sample solution for HPLC fingerprint and HPLC-ESI-MS analysis. The other residue was redissolved in 15 mL of water, and the solution was extracted by ethyl acetate (30 mL) three times. The combined water layers were further extracted by butanol (20 mL) saturated with water three times. The combined butanol layers were concentrated* in vacuo*, and the residue was redissolved in 1 mL of methanol and filtered through a 0.45 *μ*m membrane as sample solution for HPTLC analysis. In the process of selecting an optimized extraction condition, the extraction method, extraction solvent, extraction time, amount of solvent, and extraction frequency were investigated.

### 2.3. HPLC Fingerprints Analysis

Samples analysis was carried out on an Agilent 1260 HPLC series liquid chromatography system (Agilent Technologies, Santa Clara, USA), which is equipped with a binary pump, a diode-array detector, an autosampler, and a column temperature controller. Chromatographic separation was performed on a Shiseido Capcell PAK MG-C_18_ column (250 × 4.6 mm ID, 5 *μ*m; Shiseido, Japan) with the column temperature maintained at 30°C. The mobile phase consisted of acetonitrile (A) and 0.2% formic acid (B) with a linear gradient elution program (0–5 min, 1%-1% A; 5–15 min, 1%–10% A; 15–55 min, 10%–30% A; 55–65 min, 30%–35% A) at a flow rate of 1 mL/min, and the mobile phase was degassed automatically using an electronic degasser system. The injection volume was 10 *μ*L. The detector wavelength was set at 270 nm.

### 2.4. HPLC-ESI-MS Analysis

HPLC-ESI-MS analysis was performed by coupling LC to an electrospray interface and an ion trap analyzer. HPLC separation was carried out by using a Surveyor LC system (Thermo Finnigan, San Jose, CA), with a quaternary pump, continuous vacuum degasser, autosampler, and column compartment, and coupled with a variable wavelength photodiode-array detector. The conditions were the same as those of HPLC fingerprint analysis. The HPLC effluent was introduced into the ESI source in a postcolumn splitting ratio of about 3 : 1. The MS and MS^n^ analyses were acquired on an LCQ ion trap instrument (Thermo Finnigan, San Jose, CA) equipped with an Xcalibur workstation. The negative ion mode for MS analysis was selected. The operating parameters were optimized as follows: capillary voltage of 5 V, spray voltage of 2.0 kV, capillary temperature of 300°C, sheath gas flow rate at 30 (arbitrary units), and tube lens offset at 20 V. Full-scan mass spectra were recorded in the range *m*/*z* 150–1000. The isolation width of precursor ions was 1.0 Th. The HPLC and LC/MS data were acquired and processed using the Finnigan Xcalibur 1.3 software provided by the manufacturer.

### 2.5. HPTLC Analysis

5 *μ*L of the extract sample and 1 *μ*L of the reference solution were applied on 10 × 20 cm HPTLC silica gel 60 F_254_ plates (Huanghai, China) using a CAMAG (Muttenz, Switzerland) Automatic TLC Sampler (ATS4), which was controlled by Win-CATS software, and were observed by a Reprostar 3 illumination unit. The plate was developed in a twin trough chamber (10 × 20 cm) using a solvent system of ethyl acetate/butanone/formic acid/water (v/v/v/v, 4 : 3 : 1 : 1), sprayed with 1% aluminium chloride solution after developing, and then dried at 105°C for 3 minutes. The plate was examined under ultraviolet light at 366 nm, and the image was taken.

## 3. Results and Discussion

### 3.1. HPLC Fingerprint Analysis

**Figure 1:**
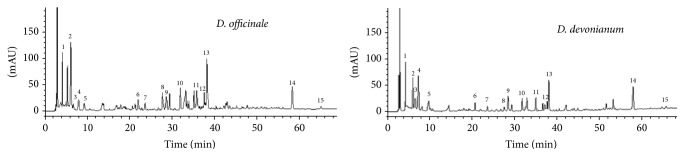
Typical HPLC chromatograms of extracts from dried stems of* D. officinale* and* D. devonianum*. Fifteen common peaks were found in* D. officinale* stems and* D. devonianum* stems, and obvious differences were shown in 20 min to 40 min in their fingerprint, indicating that the HPLC fingerprints could serve as an efficient quality control tool for differentiating* D. officinale* and* D. devonianum*.

**Figure 2:**
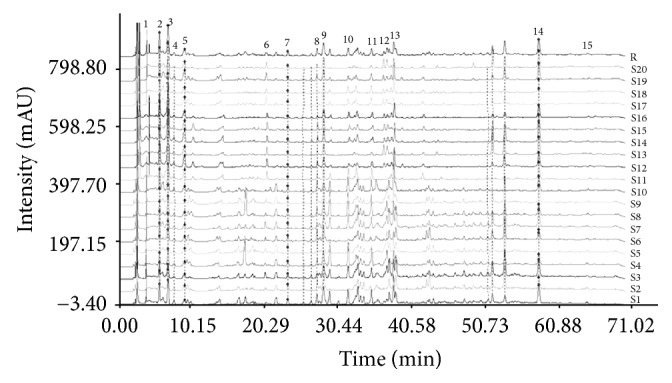
The digital standard HPLC fingerprint chromatograms of dried stems of* D. officinale* and* D. devonianum* from different regions of China. Among 15 common peaks (peak 1–peak 15), peak 14 was chosen as the reference peak to calculate RA and RRT of different peaks.* D. officinale* (S1–S10) and* D. devonianum* (S11–S20) were marked, and the sources were described in the Materials and Methods.

The HPLC fingerprint analysis method was validated in terms of specificity, precision, repeatability, and stability according to Chinese Pharmacopoeia [19]. The specificities of 15 common compounds were confirmed by their baseline separation from each other in the chromatograms (Figures 1 and 2). Precision was determined by analyzing the same sample in quintuplicate. The relative standard deviations (RSDs) of relative retention times (RRTs) and relative area (RA) for the 15 common peaks were 0.047–0.608 and 0.438–3.932%, respectively; their similarities were more than 0.99. Repeatability tests were performed with 5 independently prepared sample solutions. The RSDs of RRTs and RA of 15 common peaks were 0.027–0.921 and 1.748–3.970%, respectively, and their similarities were also over 0.99. For the stability test, the same sample solution was analyzed at 0, 2, 4, 8, and 24 h. The RSDs of RRTs and RA for the 15 common peaks were 0.036–2.123 and 0.987–3.601%, and their similarities were over 0.99. These results indicated that the developed method met the technical requirements of fingerprint analysis. The analytical method used in this study was reproducible, and the samples were stable during the test period.

Under the established HPLC condition, 10 batches of* D. officinale* stems and 10 batches of* D. devonianum* stems from various sources were analyzed, and the chromatographic fingerprint of each species was obtained, respectively.  Figure 1 shows the typical chromatograms of the stem extracts of* D. officinale* and* D. devonianum*. It can be seen that the chromatographic fingerprints of the two species exhibited both similar and different peaks with the 15 common peaks. In* D. officinale* stem, a complex chromatographic pattern having more characteristic peaks was identified, whereas* D. devonianum* stem showed less characteristic peaks, for example, showing an obvious difference with a high intensity in the 20 min to 40 min zone (Figure 1). In order to evaluate the difference and similarity on the stems of* D. officinale* and* D. devonianum*, 20 chromatograms were grouped together and subjected to Computer Aided Similarity Evaluation System for Chromatographic Fingerprint of TCM (Chinese Pharmacopoeia Commission, 2004 Version), so as to produce a digital standard fingerprint of* Dendrobium* species (Figure 2). The 15 confirmed common peaks were located in 4 and 70 min running time in all fingerprints of* D. officinale* and* D. devonianum*. Among the 15 common peaks, peak 14 was found to be generally consistent in all 20 chromatograms with reasonable height and resolution. Therefore, this peak (peak 14) was chosen as the reference peak, and RA and RRT of different peaks were calculated. The similarity analysis indicated that the similarity coefficient between* D. officinale* and* D. devonianum* was from 0.343 to 0.903 with an obvious difference, and 10 batches of* D. officinale* had similar HPLC profiles with correlation coefficients in the range of 0.873 to 0.962, while 10 batches of* D. devonianum* had similar HPLC profiles with correlation coefficients from 0.906 to 0.979. These data indicated that the chromatograms of* D. officinale* or* D. devonianum* from different sources resembled each other, respectively; however, there were specific differences between* D. officinale* and* D. devonianum* when differentiating between them.

**Figure 3:**
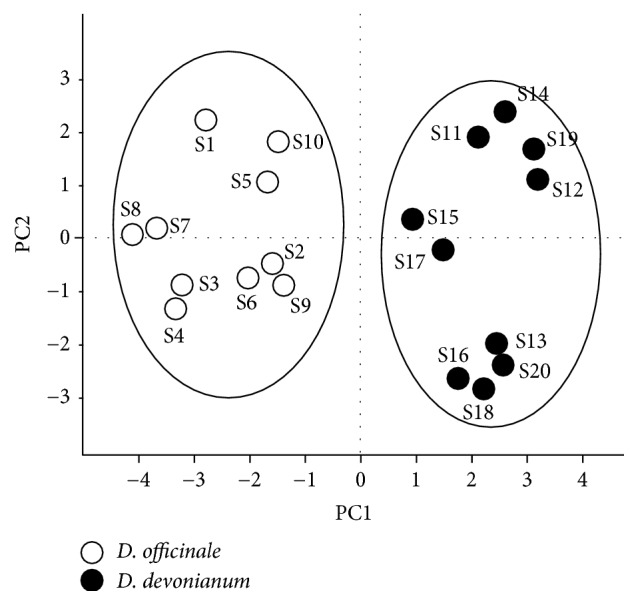
The scores plot obtained from PCA analysis of* D. officinale* and* D. devonianum*. The scores plot of PCA revealed that the 15 selected common peaks were informative to distinguish the chemical difference of* D. officinale* and* D. devonianum*, and the 20 samples were densely classified into two major groups and clearly differentiated from each other.

**Figure 4:**
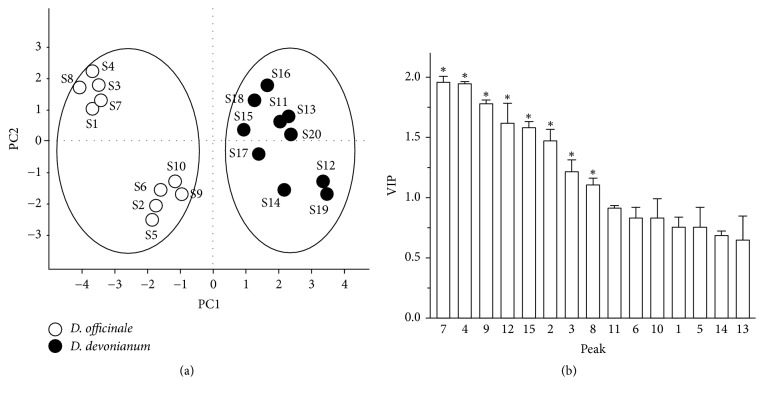
PLS-DA scores plot and VIP (variable importance plot) of* D. officinale* and* D. devonianum*. (a) The scores plot of PLS-DA revealed that* D. officinale* and* D. devonianum* could be differentiated according to the 15 selected common peaks. (b) VIP of peak 1 to peak 15 corresponding to the peaks marked in the chromatogram of HPLC fingerprints. Peaks 7, 4, 9, 12, 15, 2, 3, and 8 were all above 1.0; all the peaks were significantly different in the two classes. ^*∗*^*p* < 0.05 between* D. officinale* and* D. devonianum*.

The reliability and accuracy of chromatographic fingerprint combining similarity measure were further addressed by pattern recognition methods, including Principal Component Analysis (PCA) and Partial Least Square Discriminant Analysis (PLS-DA), which are the well-known methods in distinguishing herbal species. They were performed on the 15 common peaks of chromatographic fingerprints with 4 extracted principal components accounting for 83.8% of the total variance using SIMCA13.0.2 (Umetrics, MKS Instruments Inc., Sweden). The score plot of PCA revealed that the 15 selected common peaks were informative to distinguish chemical differences of* D. officinale* and* D. devonianum*. The 20 samples were densely classified into two major groups, which were clearly differentiated from each other (Figure 3). In the score plot of PLS-DA, the samples were correctly classified into two classes (Figure 4(a)), which was achieved in agreement with the PCA result. Moreover, the variable importance plot (VIP) indicated the variable influence on the classification of samples, showing 8 variable VIP values of peak 7, peak 4, peak 9, peak 12, peak 15, peak 2, peak 3, and peak 8. All VIP values were above 1.0, and all the peaks were significantly different in the two classes (*p* < 0.05) (Figure 4(b)). The results indicated that the 8 aforementioned peaks were the determinants for classification of* D. officinale* and* D. devonianum* stems.

### 3.2. HPLC-ESI-MS Analysis

**Figure 5:**
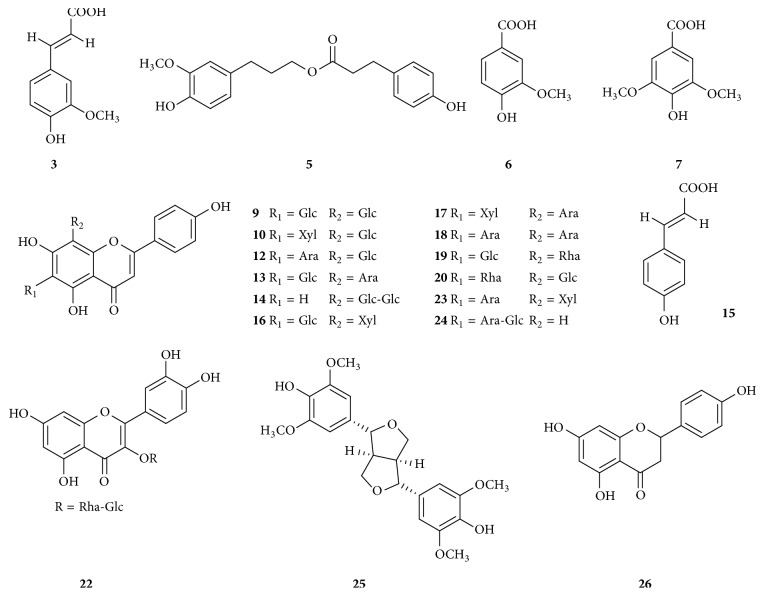
Chemical structures of constituents identified in the dried stems of* D. officinale* by HPLC-ESI-MS analysis. (3) Ferulic acid. (5) Dihydroconiferyldihydro-*p*-coumarate. (6) Vanillic acid. (7) Syringic acid. (9) Vicenin-2. (10) Apigenin-6-C-*β*-*D*-xyloside-8-C-*β*-D-glucoside. (12) Isoschaftoside. (13) Schaftoside. (14) Vitexin-2′′-O-*β*-D-glucopyranoside. (15)* p*-Hydroxycinnamic acid. (16) Vicenin-3. (17) Apigenin-6-C-*β*-D-xyloside-8-C-*β*-D-arabinoside. (18) Apigenin-6,8-di-C-*α*-L-arabinoside. (19) Violanthin. (20) Isoviolanthin. (22) Rutin. (23) Apigenin-6-C-*α*-L-arabinoside-8-C-*β*-D-xyloside. (24) Apigenin-6-C-(2′′-O-*β*-D*-*glucopyranoside)-*α*-*L*-arabinoside. (25) Syringaresinol. (26) Naringenin.

In order to identify specific chemicals in* D. officinale* stems, the chemical constituents were characterized from* D. officinale* by HPLC-ESI-MS analysis simultaneously. The structures of compounds (Figure 5) were elucidated according to the molecular weight together with multistage mass fragmentation, retention time, and relative abundance, in comparison with those found in reference compounds and literatures. The chromatogram in negative mode showed better sensitivity than that in positive mode, and a total of 26 compounds were identified, including 14 flavonoids, 5 phenolic acids, 1 lignan, and 6 other compounds (Table 1). Compounds 14, 19, 20, and 22 were reported for the first time in* D. officinale*.

Here, 14 flavonoids were further characterized, 12 of which were identified to be flavonoid glycosides. The MS/MS spectra of these compounds exhibited ions of* m/z*[M-H-60]^−^, *m*/*z*[M-H-90]^−^, and *m*/*z*[M-H-120]^−^ at different relative abundances, which were demonstrated as characteristic ions of flavonoid C-glycosides [20]. Compound 9 showed the same [M-H]^−^ ion at *m*/*z* 593, its MS/MS spectrum produced ions at *m*/*z* 503 [M-H-90]^−^ and *m*/*z* 473 [M-H-120]^−^, and it was tentatively identified as 6,8-di-C-glucosyl apigenin according to the literatures [16, 21], also named vicenin-2. Compounds 10, 12, 13, 16, and 24 produced ions at *m*/*z* 563; the fragment ions at *m*/*z* 503 [M-H-60]^−^, *m*/*z* 473 [M-H-90]^−^, and *m*/*z* 443 [M-H-120]^−^ were observed along with the sugar fragment ions (hexose and pentose) in MS/MS. According to the relative abundance of these ions, the position of C-glycosylation was confirmed in C-6 or in C-8. They were tentatively assigned as apigenin-6-C-*β*-D-xyloside-8-C-*β*-D-glucoside, isoschaftoside, schaftoside, vicenin-3 [22, 23], and apigenin-6-C-(2′′-O-*β*-D*-*glucopyranoside)-*α*-*L*-arabinoside [24, 25]. Compounds 17, 18, and 23 gave the [M-H]^−^ ions at *m*/*z* 533, and their MS^2^ fragment ions showed that their sugars were two pentoses, and thus they were tentatively characterized as apigenin-6-C-*β*-D-xyloside-8-C-*β*-D-arabinoside, apigenin-6,8-di-C-*α*-L-arabinoside, and apigenin-6-C-*α*-L-arabinoside-8-C-*β*-D-xyloside [26, 27]. Compounds 14, 19, 20, 22, and 26 were identified as vitexin-2′′-O-*β*-D*-*glucopyranoside, violanthin, isoviolanthin, rutin, and naringenin by their retention times and mass spectra with their reference standards, supported by the literatures [23, 27].

In the negative mode, the phenolic acids tended to form [M-H]^−^ ion, followed by the loss (−44 Da) of a carboxylic acid group, and provided an anion of [M-H-COO]^−^. Compound 3 produced the characteristic ion [M-H]^−^ at *m*/*z* 193.05; the product ions of the [M-H]^−^ ion gave the fragment ions [M-H-CH_3_]^−^ at *m*/*z* 175.07 and [M-H-COO]^−^ at *m*/*z* 148.95, and thus compound 3 was assigned as ferulic acid. Compounds 5, 6, 7, and 15 were characterized by the same pattern as dihydroconiferyldihydro-*p*-coumarate, vanillic acid, syringic acid, and* p*-hydroxycinnamic acid [28–34]. The product ions of the [M-H]^−^ ion (compound 25) at *m*/*z* 417.02 gave the fragment ions [M-H-CH_3_]^−^ at *m*/*z* 402.12, [M-H-CH_3_-CH_3_]^−^ at *m*/*z* 387.12, and [M-H-C_13_H_16_O_4_]^−^ at *m*/*z* 181.12, suggesting the presence of syringaresinol [35]. The corresponding structures of the other 6 fragment ions have not yet been found, and we need to do more research for their identification in the future.

In order to detect the specific compound from* D. officinale*, the same mass spectrometry conditions were applied to analyze the compound difference of* D. devonianum* by selected ion monitoring (SIM) of mass spectrometry scanning mode, in which only a limited mass-to-charge ratio range is detected by the instrument. The results showed that the molecular ion peak of the [M-H]^−^ ion at *m*/*z* 577.07 was also found in* D. devonianum*; however, its retention time was 46.16 min and fragmentation ions in MS/MS were at *m*/*z* 457.08(4), 413.08(5), 311.08(4), and 269.14(100), which were totally different from those of [M-H]^−^ ion at *m*/*z* 577.07 of violanthin and isoviolanthin in* D. officinale*. Thus, violanthin and isoviolanthin are the specific components of* D. officinale,* which could be used as reference substances to distinguish* D. officinale* from* D. devonianum*. Meanwhile, schaftoside was also identified from the stem of* D. devonianum*, which could be used as a common reference to evaluate* D. officinale* and* D. devonianum*.

### 3.3. HPTLC Fingerprints

**Figure 6:**
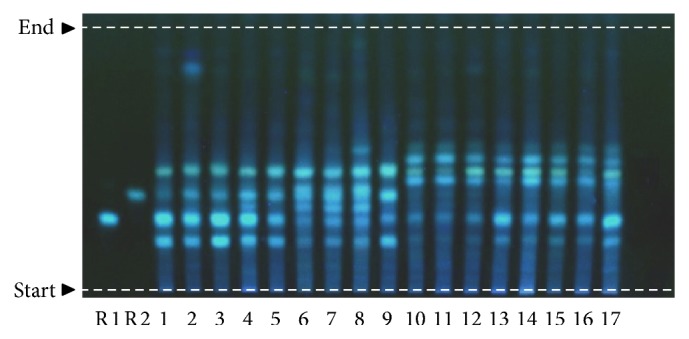
HPTLC chromatogram of* D. officinale* and* D. devonianum* stems. Schaftoside (R1) and violanthin (R2) were used as markers. Nine batches of* D. officinale* (tracks 1–9) and eight batches of* D. devonianum* (tracks 10–17) were shown.

To investigate the presence and compare the difference of flavonoids in the stems of* D. officinale* and* D. devonianum* by a convenient and effective method, an optimized HPTLC method was further developed and validated with good reproducibility, selectivity, and durability. Using violanthin and schaftoside as reference substances, good separation was attained with the desired *R*_*f*_-value range (0.2–0.8) for all bands, 0.26 for schaftoside, and 0.35 for violanthin on a silica gel backed HPTLC plate (silica gel 60 F_254_, 10 × 20 cm) using a mobile phase of ethyl acetate/butanone/formic acid/water (v/v/v/v, 4 : 3 : 1 : 1) [36]. Room temperature and relative humidity at the time of development were 25.0 ± 2.0°C and 45.0 ± 2.0%, respectively. HPTLC chromatograms of* D. officinale* and* D. devonianum* are shown in Figure 6. The observation at 366 nm allowed a clear visualization of reference compounds and other bands on the chromatogram, and all bands were well separated, symmetrical, and nontrailed. In 9 batches of* D. officinale* (tracks 1–9), violanthin was easily found with *R*_*f*_ value of 0.35; however, it was not observed in eight batches of* D. devonianum* (tracks 10–17), while schaftoside (*R*_*f*_ = 0.26) was revealed as a common component in both species. The results indicated that violanthin could be a specific compound of* D. officinale* by the developed HPTLC method in differentiating* D. officinale* and* D. devonianum.*

## 4. Conclusion

In the present work, an effective HPLC fingerprint analysis method for differentiation and identification of* D. officinale* and* D. devonianum* was developed, with the chromatogram and pattern recognition of HPLC fingerprints: the two species were successfully distinguished from each other. Using HPLC coupled with ion trap mass spectrometry, a tentative identification of 26 compounds of* D. officinale* could be proposed, including 15 flavonoids, 5 phenolic acids, and 1 lignan. The flavonoids detected were mainly composed of apigenin glycoside derivatives. In addition, violanthin and isoviolanthin were detected in* D. officinale* whereas they were not detected in* D. devonianum*. A rapid HPTLC analysis was developed and successfully applied for detecting and identifying the stems of* D. officinale* and* D. devonianum* with their common compound, schaftoside, and specific compounds of violanthin, as in* D. officinale*. Using violanthin as a specific compound of* D. officinale*,* D. devonianum* could be differentiated by the method of HPTLC. As the results shown here, the developed methods could be used as a combined quality control strategy effectively and efficiently for* D. officinale* and* D. devonianum*.

## Supplementary Material

Supporting Fig. 1 Graphical abstract.
Supporting Fig. 2 LC-MS/MS spectrums of compounds 9, 10, 12, 13, 16, and 24 isolated from the stem of *D. officinale* (negative ion mode).
Supporting Fig. 3 LC-MS/MS spectrums of compounds 17, 18, 23, 14, 19, and 20 isolated from the stem of *D. officinale* (negative ion mode).
Supporting Fig. 4 LC-MS/MS spectrums of compounds 3, 5, 6, 7, 15, and 25 isolated from the stem of *D. officinale* (negative ion mode).
Supporting Fig. 5 LC-MS chromatograms (1) LC-MS spectrums (2) and LC-MS^2^ spectrums (3) of violanthin (A) and isoviolanthin (B) from the stem of *D. officinale* (negative ion mode).
Supporting Fig. 6 The MS spectra and the characteristic fragmentation pathway of violanthin and isoviolanthin. Violanthin (A; compound 19) and isoviolanthin (B; compound 20) were shown here.

## Figures and Tables

**Table 1:** Characterization of compounds from the extract of *D. officinale* stems by HPLC-ESI-MS (in negative mode). A total of 26 compounds were identified, including 14 flavonoids, 5 phenolic acids, 1 lignan, and 6 other compounds from the stems of *D. officinale*.

Compound	*T*_*R*_/min	MS[M-H]^−^ *m/z* observed	MS^2^ *m/z* observed	Formula	Identification
1	3.20	165.07	147.02(100), 129.00(60), 118.87(27)	*∗∗*	To be identified
2	3.25	167.04	149.08(100), 123.29(10), 85.88(13), 75.09(80)	*∗∗*	To be identified
3	3.67	192.96	177.09(43), 148.93(51)	C_10_H_10_O_4_	Ferulic acid
4	16.84	286.91	268.91(20), 241.00(100), 135.11(10)	*∗∗*	To be identified
5	18.86	329.01	285.16, 165.08(100), 121	C_19_H_22_O_5_	Dihydroconiferyldihydro-*p*-coumarate
6	18.74	167.05	152.03(30), 123.07(100), 108.11(5)	C_8_H_8_O_4_	Vanillic acid
7	20.66	197.05	182.07(100), 153.09(50), 137.86	C_9_H_10_O_3_	Syringic acid
8	24.30	293.03	131.07(100)	*∗∗*	To be identified
9	30.00	593.48	533.22(8), 503.15(22), 473.13(66), 353.17(6)	C_27_H_30_O_15_	Vicenin-2
10	32.05	563.48	503.17, 473.10(100), 443.12(60), 353.14(13)	C_26_H_28_O_14_	Apigenin-6-C-*β*-*D*-xyloside-8-C-*β*-D-glucoside
11	33.68	612.84	553(40), 539.16(100), 492.96(40), 459.01(30), 451.06(50), 293.28(10)	*∗∗*	To be identified
12	33.80	563.18	503.20(60), 473.10(100), 443.09(64), 353.20(8)	C_26_H_28_O_14_	Isoschaftoside
13	35.93	563.58	503.20(22), 473.17(47), 443.16(41), 353.14(7)	C_26_H_28_O_14_	Schaftoside
14	36.57	593.67	473.10(10), 413.12(100), 293.17(6)	C_27_H_30_O_15_	Vitexin-2′′-*O*-*β*-*D*-glucopyranoside
15	36.61	163.08	163.16, 119.29	C_9_H_8_O_3_	*p*-Hydroxycinnamic acid
16	37.12	563.21	503(4), 473.17(22), 443.13(20), 353.03(6)	C_26_H_28_O_14_	Apigenin-6-C-*β*-*D*-glucoside-8-C-*β*-*D*-xyloside
17	38.04	533.58	515.15(30), 473.24(40), 443.15(100), 383.08(10)	C_25_H_26_O_13_	Apigenin-6-C-*β*-*D*-xyloside-8-C-*β*-*D*-arabinoside
18	38.38	533.17	515.18(22), 473.18(40), 443.16(80)	C_25_H_26_O_13_	Apigenin-6,8-di-C-*α*-*L*-arabinoside
19	38.61	577.07	559.2(28), 503.02(20), 487.20(50), 473(30), 457.15(100), 383.20(23)	C_27_H_30_O_14_	Violanthin
20	38.82	577.22	559.2(42), 503.19(78), 473.15(76), 457.13(100), 383.15(40)	C_27_H_30_O_14_	Isoviolanthin
21	39.37	596.24	434.01(100)	*∗∗*	To be identified
22	39.62	609.04	301.05(100)	C_27_H_30_O_16_	Rutin
23	41.22	533.55	515.15(26), 473.16(46), 443.15(72)	C_25_H_26_O_13_	Apigenin-6-C-*α*-L-arabinoside-8-C-*β*-*D*-xyloside
24	44.03	563.10	473.13(15), 383.16(100)	C_26_H_28_O_14_	Apigenin-6-C-(2′′-O-*β*-D-glucopyranoside)-*α*-*L*-arabinoside
25	45.02	417.02	402.12(40), 387.16(12), 181.12(100), 166.09(60)	C_22_H_26_O_8_	Syringaresinol
26	66.21	271.23	177.15(15), 151.08(80)	C_15_H_12_O_5_	Naringenin

*T*_*R*_: retention time. *∗∗*: to be identified.
