# Supplementary material for: Chemical Differentiation of Dendrobium officinale and Dendrobium devonianum by Using HPLC Fingerprints, HPLC-ESI-MS, and HPTLC Analyses
Source: Evid Based Complement Alternat Med. 2017 Jul 10;2017:8647212. doi: 10.1155/2017/8647212 (PMC5523191; doi:10.1155/2017/8647212)
Supplement: Supplementary file 1 — Supporting Fig. 1 Graphical abstract. Supporting Fig. 2 LC-MS/MS spectrums of compounds 9, 10, 12, 13, 16, and 24 isolated from the stem of D. officinale (negative ion mode). Supporting Fig. 3 LC-MS/MS spectrums of compounds 17, 18, 23, 14, 19, and 20 isolated from the stem of D. officinale (negative ion mode). Supporting Fig. 4 LC-MS/MS spectrums of compounds 3, 5, 6, 7, 15, and 25 isolated from the stem of D. officinale (negative ion mode). Supporting Fig. 5 LC-MS chromatograms (1) LC-MS spectrums (2) and LC-MS2 spectrums (3) of violanthin (A) and isoviolanthin (B) from the stem of D. officinale (negative ion mode). Supporting Fig. 6 The MS spectra and the characteristic fragmentation pathway of violanthin and isoviolanthin. Violanthin (A; compound 19) and isoviolanthin (B; compound 20) were shown here. [file 8647212.f1.pdf]

Supporting figures

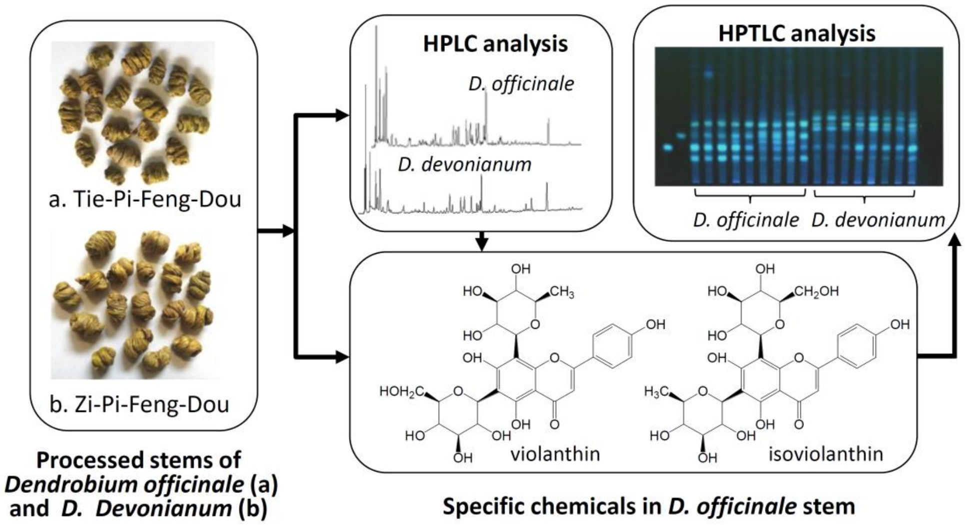

Supporting Fig. 1 Graphical abstract

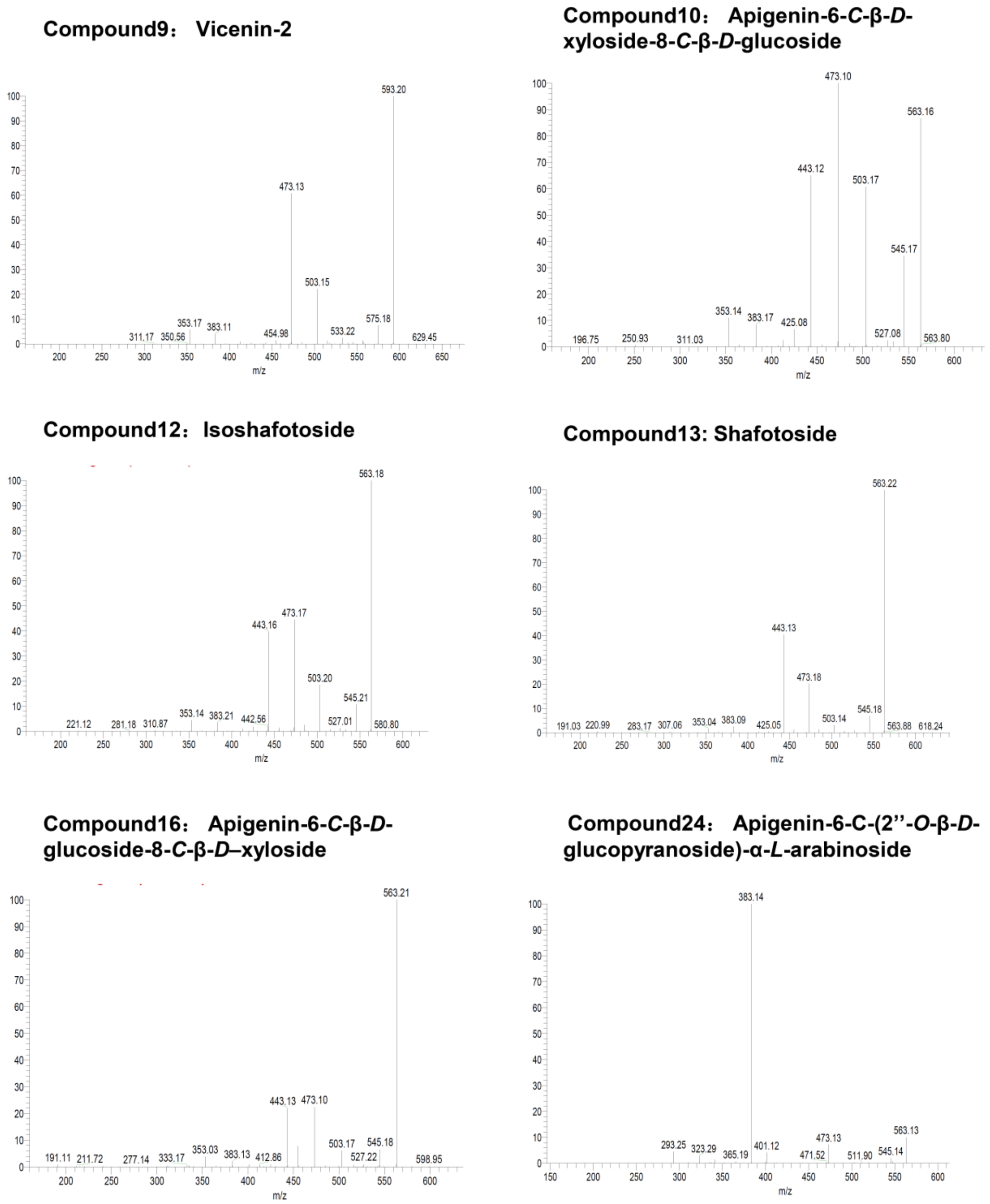

Supporting Fig. 2 LC-MS/MS spectrums of vicenin-2, apigenin-6-*C*-β-*D*-xyloside-8-*C*-β-*D*-glucoside, isoshafotoside, shafotoside, apigenin-6-*C*-β-*D*-glucoside-8-*C*-β-*D*–xyloside and apigenin-6-*C*-(2’’-*O*-β-*D*-glucopyranoside)-α-*L*-arabinoside from the stem of *D. officinale*（negative ion mode）.

Compound 17: Apigenin-6-*C*-β-*D*-xyloside-8-*C*-β-*D*-arabinoside

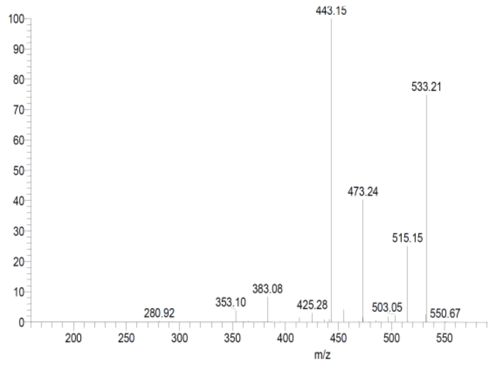

Compound 18: Apigenin-6,8-*di*-*C*-α-*L*-arabinoside

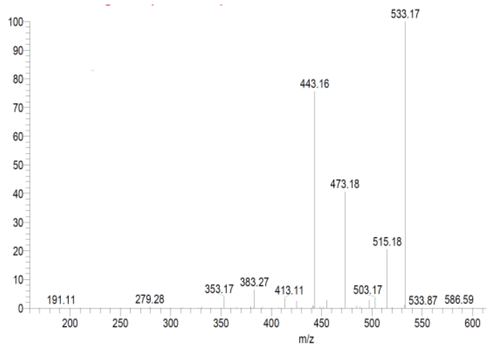

Compound 23: Apigenin-6-*C*-α-*L*-arabinoside-8-*C*-β-*D*-xyloside

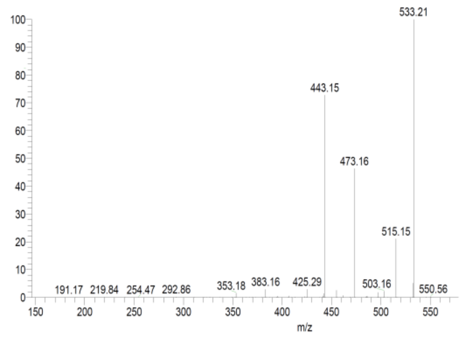

Compound 14: Vitexin 2''-*O*-β-*D*-glucopyranoside

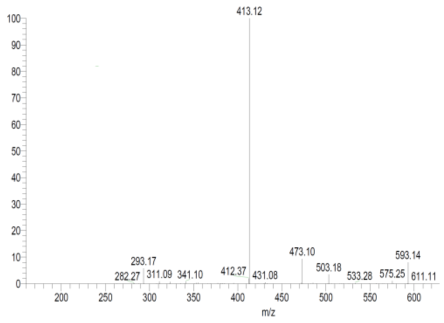

Compound 19: Violanthin

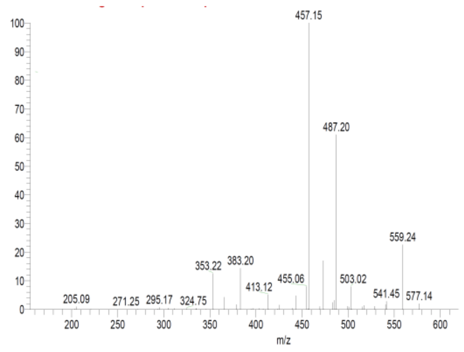

Compound 20: Isoviolanthin

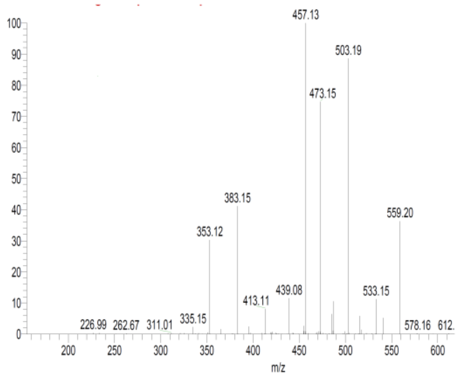

Supporting Fig.3 LC-MS/MS spectrums of apigenin-6-*C*-β-*D*-xyloside-8-*C*-β-*D*-arabinoside, apigenin-6,8-*di*-*C*-α-*L*- arabinoside, apigenin-6-*C*-α-*L*-arabinoside-8-*C*-β-*D*-xyloside, vitexin 2''-*O*-β-*D*-glucopyranoside, violanthin and isoviolanthin from the stem of *D. officinale*（negative ion mode）**.**

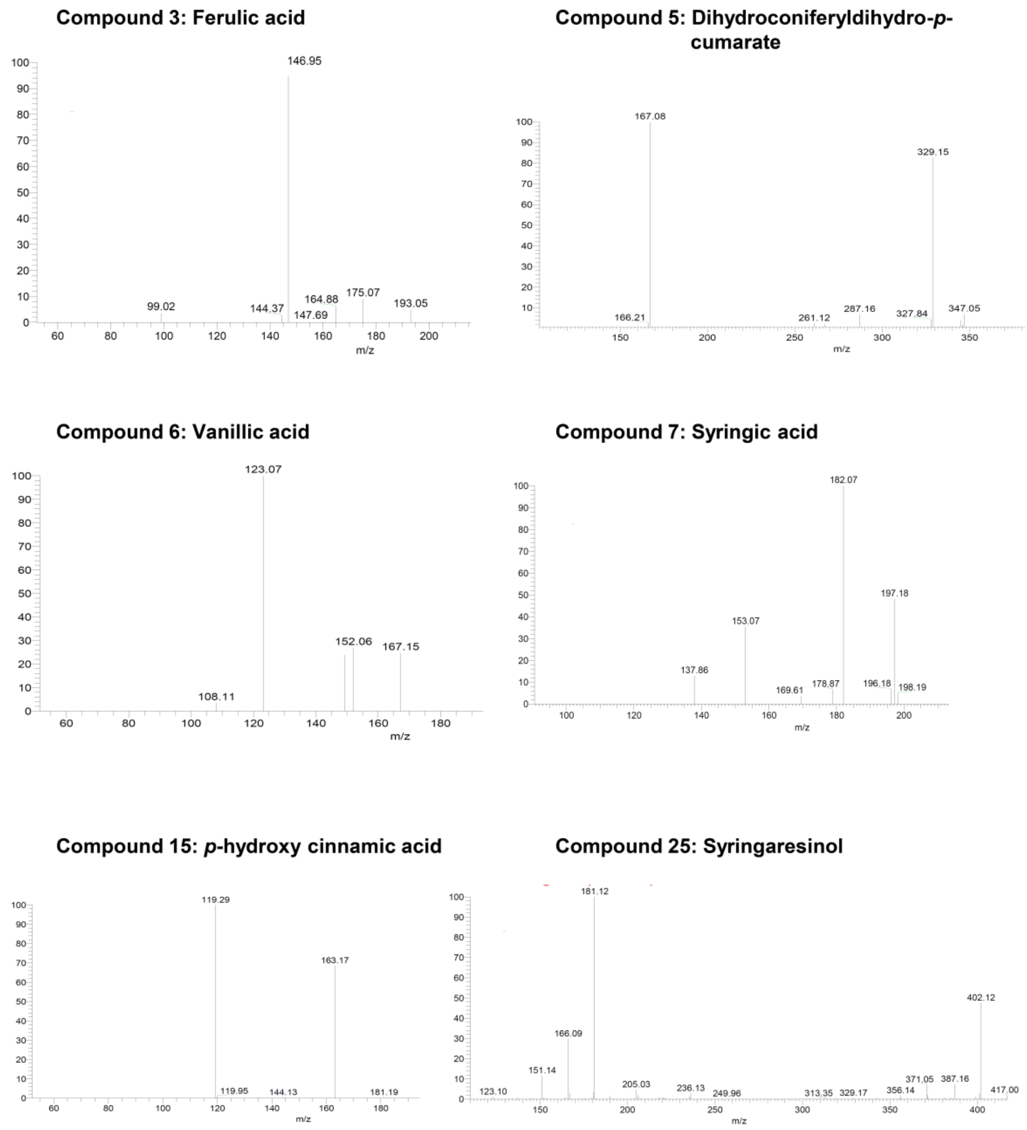

Supporting Fig. 4 LC-MS/MS spectrums of ferulic acid, dihydroconiferyldihydro-*p*-cumarate, vanillic acid, syringic acid, *p*-hydroxycinnamic acid and syringaresinol from the stem of *D. officinale*（negative ion mode）.

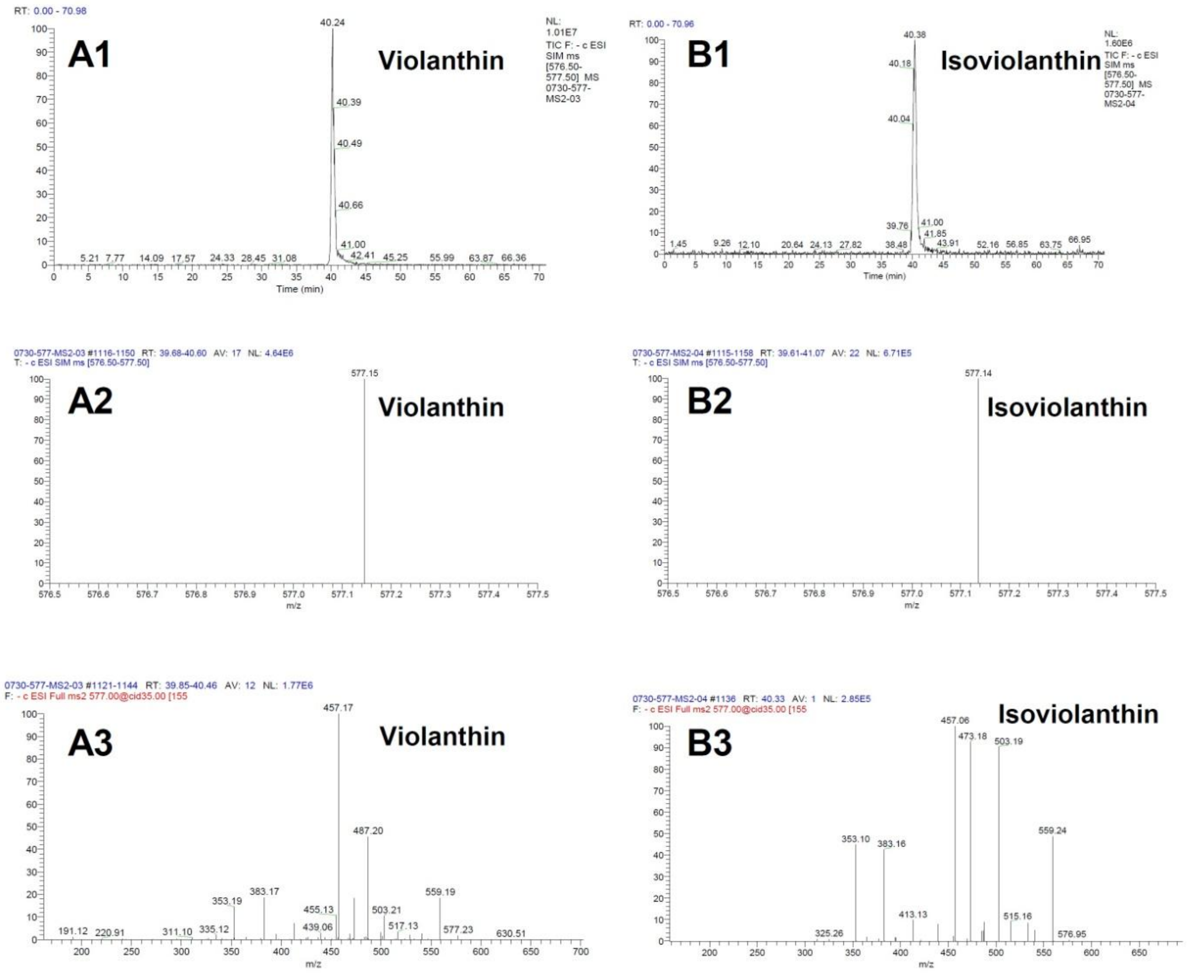

Supporting Fig.5 LC-MS chromatograms (1) LC-MS spectrums (2) and LC-MS^2 spectrums (3) of violanthin (A) and isoviolanthin(B) from the stem of *D. officinale*（negative ion mode）

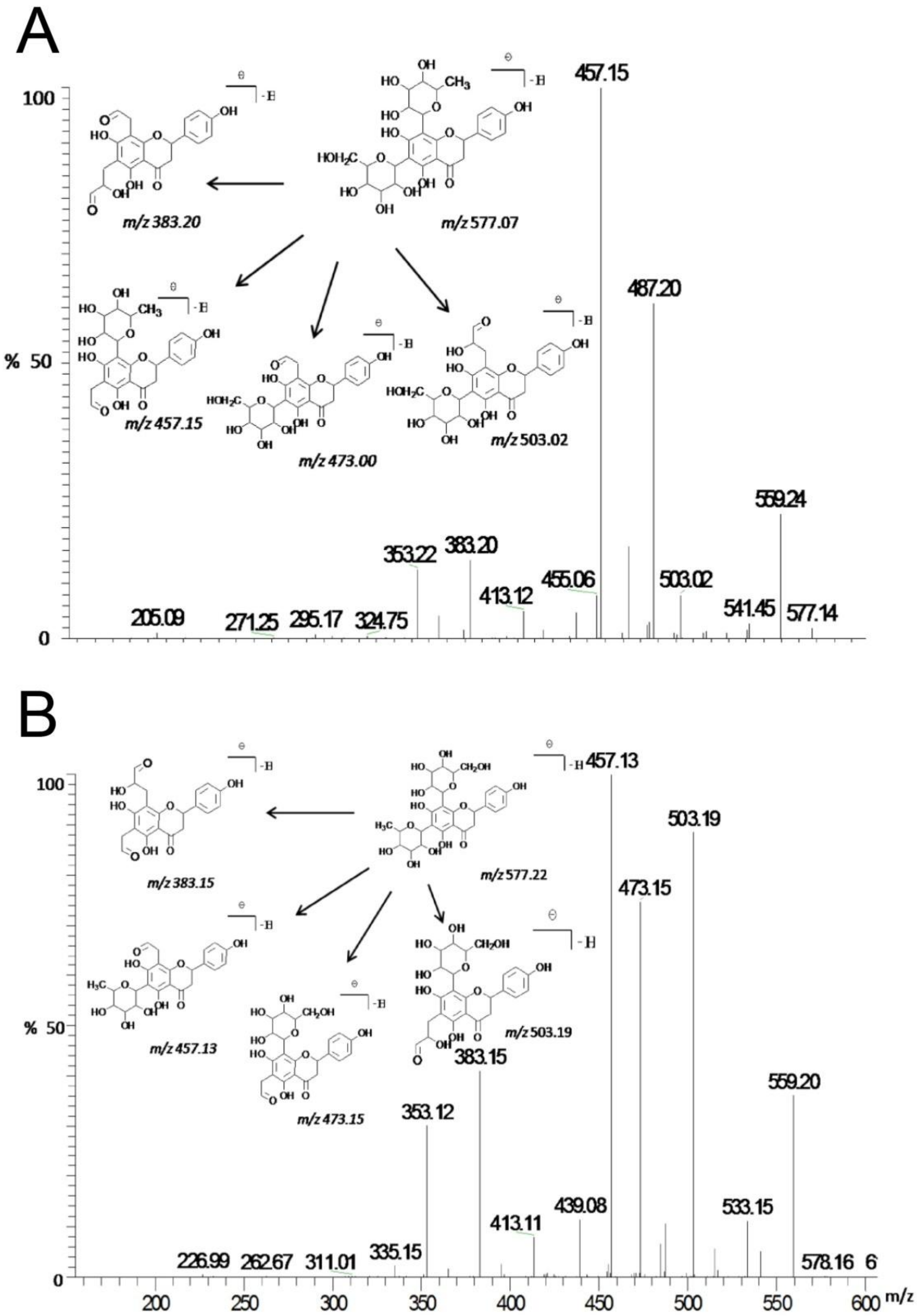

Supporting Fig.6 The MS spectra and the characteristic fragmentation pathway of violanthin and isoviolanthin. Violanthin (**A**; compound 19) and isoviolanthin (**B**; compound 20) were shown here.
